# Paradoxical cold conditions during the medieval climate anomaly in the Western Arctic

**DOI:** 10.1038/srep32984

**Published:** 2016-09-09

**Authors:** Vincent Jomelli, Timothy Lane, Vincent Favier, Valerie Masson-Delmotte, Didier Swingedouw, Vincent Rinterknecht, Irene Schimmelpfennig, Daniel Brunstein, Deborah Verfaillie, Kathryn Adamson, Laëtitia Leanni, Fatima Mokadem, ASTER Team, Georges Aumaître, Didier L. Bourlès, Karim Keddadouche

**Affiliations:** 1 Université Paris 1 Pantheon-Sorbonne, CNRS Laboratoire de Géographie Physique, 92195 Meudon, France; 2 School of Natural Sciences and Psychology, Liverpool John Moores University, Liverpool L3 3AF, UK; 3 Univ. Grenoble Alpes, LGGE, F-38041 Grenoble, CNRS, France; 4 LGGE, F-38041 Grenoble, France; 5 LSCE/IPSL, UMR 8212 (CEA-CNRS-UVSQ), Université Paris Saclay, CEA Saclay, Gif-sur-Yvette 91191, France; 6 EPOC, Universite Bordeaux 1, Allée Geoffroy Saint-Hilaire, Pessac 33615, France; 7 Aix-Marseille Université, CEREGE CNRS-IRD UMR 34, Collège de France, 13545 Aix-en-Provence, France; 8 School of Science and the Environment, Manchester Metropolitan University, Manchester M1 5GD, UK

## Abstract

In the Northern Hemisphere, most mountain glaciers experienced their largest extent in the last millennium during the Little Ice Age (1450 to 1850 CE, LIA), a period marked by colder hemispheric temperatures than the Medieval Climate Anomaly (950 to 1250 CE, MCA), a period which coincided with glacier retreat. Here, we present a new moraine chronology based on ^36^Cl surface exposure dating from Lyngmarksbræen glacier, West Greenland. Consistent with other glaciers in the western Arctic, Lyngmarksbræen glacier experienced several advances during the last millennium, the first one at the end of the MCA, in ~1200 CE, was of similar amplitude to two other advances during the LIA. In the absence of any significant changes in accumulation records from South Greenland ice cores, we attribute this expansion to multi-decadal summer cooling likely driven by volcanic and/or solar forcing, and associated regional sea-ice feedbacks. Such regional multi-decadal cold conditions at the end of the MCA are neither resolved in temperature reconstructions from other parts of the Northern Hemisphere, nor captured in last millennium climate simulations.

During the last millennium, the Northern Hemisphere experienced mild temperatures during the Medieval Climate Anomaly (MCA, ~950 to 1250 CE) and about 0.5 °C cooler temperatures during the Little Ice Age (LIA, ~1450 to 1850 CE)^1^. Due to their sensitivity to summer temperature, mid-latitude mountain glaciers such as in the Alps retreated during the MCA and experienced major advances during the LIA, the largest advances of the past few millennia^2^,^3^ due to a multi-millennial cooling trend^1^,^4^,^5^.

In Greenland however, the fluctuations of the ice sheet and its peripheral glaciers remain poorly constrained. Recent studies of the Greenland ice sheet (GrIS), based on terrestrial cosmic-ray exposure (CRE) moraine dating and lake sediment analyses, revealed asynchronous advances during the LIA^2^, that are thought to be attributable to long ice sheet response time, regional variation in ocean and atmosphere temperature patterns^6^,^7^,^8^, and different termini types (terrestrial or marine)^9^,^10^. For instance, in West Greeland, the marine-terminating Jakobshavn Isbræ advanced at 1800 CE, and had retreated by 1850 CE^9^,^11^. A 19^th^ century maximum extent is also reported from the Qajuuttap Sermia land margin of the southern GrIS^12^. However, land-based sectors of the GrIS north of Jakobshavn advanced at 1400–1700 CE, and retreated after 1820 CE^13^. The land-terminating portion of GrIS south of Jakobshavn reached its maximum position during the twentieth century^10^.

Fluctuations of Greenland mountain glaciers from the end of the 19^th^ century have been largely documented from drawings and expedition photographs^14^,^15^. Studies covering the whole LIA period are limited. Absolute dating records are rare, and largely derived from Scoresby Sund, East Greenland. One moraine of Gurrenholm Dal glacier has been dated to c. 1200–1700 CE using four CRE ages^16^. In this part of East Greenland region, another study focused on an unweathered and unvegetated moraine in front of the Bregne ice cap. The CRE ages of the 9 samples range from 0.74–9.60 kyr. The authors suggest that the youngest age 0.74 kyr (1250 CE) may represent the timing of moraine formation^17^. Close to Bregne ice cap, ref. 18 interpret the recently exposed fossil vegetation (radiocarbon ages) record as representing warm times and suggested that the Istorvet ice cap expanded prior to 1150 CE and subsequently retreated after 1660 CE. Recently, a moraine of Uigordleq Lake valley, West Greenland was dated based on four ^10^Be ages to 1130 ± 40 CE^19^.

Collectively, these studies show contrasted glacier advances. Unfortunately, moraine chronologies are scarce and it is not possible to unequivocally identify the LIA signal or determine any climatic drivers of glacier change^16^,^17^,^18^,^19^ mainly due to widespread isotopic inheritance that makes it hard to interpret those records.

Here, we present a new cosmic-ray exposure moraine chronology of an outlet glacier of the Lyngmarksbræen ice cap (69.36°N; 53.51°W; 32 km^2^ in 2015), Disko Island, West Greenland (Fig. 1), which flows northeast from a high altitude basalt plateau (up to 995 m above sea level a.s.l.). This land-terminating glacier is not influenced by calving processes induced by oceanic feedbacks, and variations are driven by summer temperature and accumulation making it well-suited to reconstruct past climate conditions.

Combining our new chronology with other West Arctic records (90–50°W; 60–80°N), we investigate the following questions: (i) when did maximum glacial extents occur during the last millennium? (ii) which climate factors drove these advances? Through comparison of our results with an ensemble of ocean-atmosphere general circulation model (OAGCM) simulations from the Paleoclimate Modelling Intercomparison Project PMIP3^20^,^21^, new external forcing^22^ and temperature reconstructions^1^,^4^,^5^, we then investigate the consistency of glacier-inferred West Arctic climatic changes with Northern Hemisphere temperature reconstructions and ensemble simulations spanning the last millennium.

Four moraines (hereafter M1–4) were mapped in the field and deemed suitable for CRE dating. Samples were taken from the upper surfaces of boulders (n = 14) on the crests of each moraine (Method and extended data Table S1). Whole rock samples were prepared for *in situ*^36^Cl dating at CEREGE, France (Methods). Moraine ages were calculated following ref. 23, using the ^36^Cl production parameters outlined below (Methods and extended data Tables S1–S4) and the time-invariant scaling method by ref. 24. Applying other scaling models yields insignificant age differences due to the high latitude of the study site (<5%). Individual ages refer to the sampling year 2013. Uncertainties are reported at 1σ level and were calculated through full propagation of analytical and production rate errors. The moraine ages are reported as arithmetic means of the respective population of boulder ages and their uncertainties include the standard deviation and the mean of the individual age uncertainties (including analytical and production rate errors), added by propagation in quadrature. All but the oldest ages are reported in years CE. Ages from M1, M2, and M3 are all internally consistent based on Chauvenet’s criterion test.

## Results

**Figure 1:**
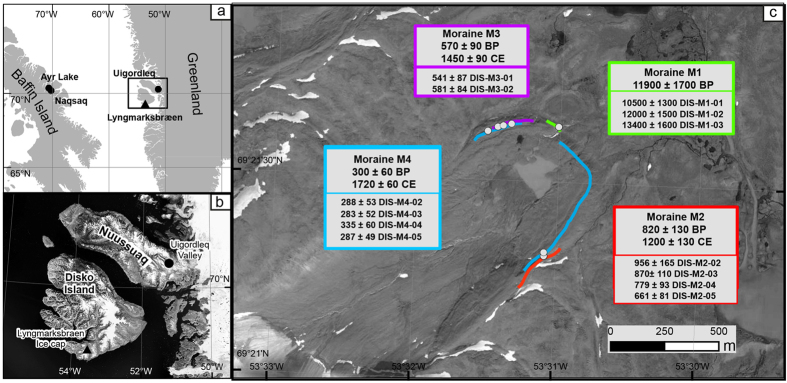
The Lyngmarksbræen glacier and studied sites. (**a**) Location of existing ^10^Be moraine chronologies in Baffin and Greenland spanning the MCA (black circles)^19^ and our new ^36^Cl moraine record (black triangle). (**b)** Zoom on the location of the Lyngmarksbræen glacier valley on Disko Island (black triangle), and the Uigordleq valley moraine in west Greenland^19^ (black circle). (**c)** Map of the Lyngmarksbræen glacier, showing dated moraines (see Methods) and the location of ^36^Cl samples (white circles). The reported age uncertainties account for standard deviation, analytical and production rate uncertainties. Satellite image by DigitalGlobe, Inc. Copyright^©^ 201X DigitalGlobe, Inc. www.digitalglobe.com. The figure was created and ArcGis 10.2 Arcinfo single use. http://www.esri.com and Adobe Illustrator CS5 V.15.0.2.

The maximum extent of Lyngmarksbræen glacier is indicated by a 30 m long, 5 m high moraine remnant (M1) located 1.7 km from the present ice margin at 416 m a.s.l. (Fig. 1). Ages from M1 (n = 3) range from 10.5 ± 1.3 kyr to 13.4 ± 1.6 kyr, and yield a mean ^36^Cl age of 11.9 ± 1.7 kyr. Moraines M2, M3 and M4 are distinct moraine ridges, found ~50 m upslope of M1 (Fig. 1; Extended data Fig. S1; Tables S1–4). M2 forms a distinct lateral arcuate ridge of about 20 m high and about 60 m long. M3 and M4 form longer nested ridges. The frontal vallum is formed by the nesting of M2 and M3 and possibly on the right side of the valley by the nesting of M2 and M4. Two other small (<2 m high) discontinuous ridges were identified between M2 and M4 and upslope from M4 but were judged to be too small and fragmentary to be robustly dated. Four samples collected on the lateral part on M2 yield ^36^Cl ages between 660 ± 80 yr and 960 ± 170 yr with a mean age of 1200 ± 130 CE. Two samples collected on the left lateral part of moraine M3 result in ^36^Cl ages of 540 ± 90 yr and 580 ± 80 yr with a mean of 1450 ± 90 CE. The ^36^Cl ages of four samples collected from M4 range from 280 ± 50 yr to 340 ± 60 yr with a mean age of 1720 ± 60 CE. One age was rejected from the M4 dataset as an outlier based on the Chauvenet’s criterion test: DIS-M4-01: 4.67 ± 0.54 kyr. This moraine boulder likely has been pre-exposed to cosmic radiation and was reworked during the last glacier advance. Excluding this one outlier, each boulder age population is internally consistent, and all mean moraine ages agree with the stratigraphic order of moraine depositions, i.e. the oldest moraines are located furthest from the present day ice margin (Fig. 1; extended Fig. S1). This substantially supports the precision of our moraine chronology, which represents one of the first datasets to produce such young ages from ^36^Cl surface exposure dating.

Our moraine chronology indicates that following the deposition of M1 during the Late Glacial/Holocene transition around 11.9 kyr, Lyngmarksbræen glacier reached similar extents during the last millennium. The advance recorded by M2 occurred at the end of the MCA, prior to ~1200 CE (M2). Moraines that might have been deposited after ~1200 CE were wiped out by LIA glacier advances, and only the M2 MCA moraine segment survived. During the LIA, the glacier margin experienced extensive re-advances or re-stabilizations similar to the ~1200 CE extent at ~1450 CE (M3) and ~1720 CE (M4), before beginning the 1 km retreat toward its present margin.

**Figure 2:**
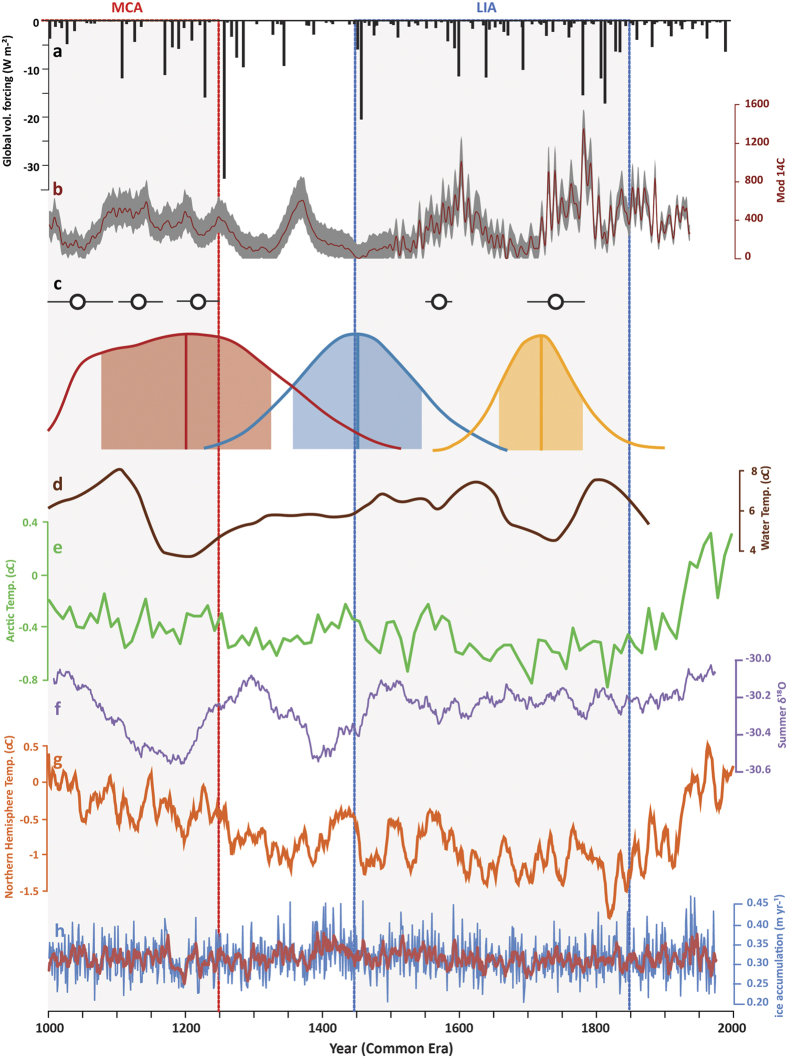
Changes in the Lyngmarksbræen glacier compared with information on past climate variability from proxy records. (**a**) Volcanic forcing (W/m^2^) estimated from polar ice core sulfate aerosol deposition records used in PMIP^21^ (extended Table S5) updated with data from ref. 22. (**b**) Total solar irradiance^21^. **(c**) ^10^Be moraine ages (circles) from Baffin Island and West Greenland^19^ and ^36^Cl moraine ages (density functions) from this study. The density functions are generated from the individual boulder ages with their analytical errors. Vertical lines represent arithmetic means, and colored bands are the age uncertainty accounting for standard deviation, analytical and production rate uncertainties. (**d**) Alkenone-based lake water temperature reconstruction from near Kangerlussuaq, West Greenland^28^. (**e**) Arctic reconstructed summer temperatures^4^. (**f)** Summer d18O from Greenland ice cores^31^. **(g**) Mean northern Hemisphere reconstructed temperature^1^. (**h)** Mean snow accumulation stack from DYE-3, Crete, GRIP, and NGRIP^25^. The periods of the Medieval Climate Anomaly (MCA: 950–1250 CE) and the Little Ice Age (LIA: 1300–1850 CE) are shown with grey bands and limited with the red and blue vertical lines, respectively. The figure was generated with Adobe Illustrator CS5 V.15.0.2.

A major glacier advance during the warm MCA appears paradoxical. We therefore review the published ^10^Be moraine chronologies in West Arctic regions above 60°N. In Baffin Bay^19^, at least three moraine remnants were recently dated to the MCA (Figs 1 and 2). The ^36^Cl age of the Lyngmarksbræen M2 moraine (1200 ± 130 CE) overlaps with the ^10^Be ages of these three Baffin Bay moraines (1040 ± 60 yr CE, 1130 ± 30 yr CE, 1220 ± 30 yr CE). Another moraine in Uigordleq valley in West Greenland (Fig. 1) was also dated to 1130 ± 40 yr CE^19^. In the same study, ^10^Be ages of two LIA moraines (1570 ± 20 CE, 1740 ± 40 CE) are very similar to our two ^36^Cl moraine ages coinciding with the LIA (1450 ± 90 CE and 1720 ± 60 CE). We conclude that West Arctic glaciers experienced substantial glacier advances during the MCA to LIA period with MCA glacier extents similar to that of the ensuing LIA. This contrasts with patterns of glacier behaviour observed in mid-latitude mountains e.g. the Alps and Scandinavia^2^,^3^.

## Discussion

The results from the recent study in Baffin Bay and ours are in remarkable agreement, implying that in total four mountain glaciers in the Baffin Bay region experienced near-synchronous large glacier advance during the Late Holocene. This suggests that these four glaciers responded to a common regional climate forcing rather than to local climatic or non-climatic drivers.

Mountain glaciers are sensitive to both summer temperature and precipitation changes. Even if they may not be representative of variations in coastal north-western Greenland^25^, snow accumulation records from Greenlandic ice cores^26^ show multi-decadal variations during the last millennium (Fig. 2). However, given the small magnitude (<10%) and the asynchrony of these accumulation changes between ice core records, we attribute variations in glacier extent to summer temperature variations. This is in agreement with the physical processes driving glacier ablation in West Greenland today^27^.

The moraine records in Disko and Baffin Bay are therefore interpreted to indicate several cool multi-decadal summer episodes during the MCA that were possibly as cold as the cold peaks of the LIA, leading to MCA glacier advances close to those of the LIA. This pattern is consistent with evidence of a cold interval from 1150 to 1220 CE identified in reconstruction of lake water summer temperatures south of the Disko region^28^; sea surface temperatures from Disko Bugt^29^ (Fig. 2) and regional sea ice concentration^30^. At a larger scale, temperature-sensitive Greenland ice core records show coherently milder episodes during the MCA (from the 9^th^ to the 12^th^ centuries) than during the LIA, with nonetheless contradictory results for the timing of the warmest and coldest phases, and contrasting patterns for summer versus winter signals^31^,^32^. Cold conditions during the MCA contrast with evidence for mild conditions in the late 13^th^ century both at lower latitudes and to the east of Greenland^2^,^33^ and highlights the complex spatio-temporal structure of the late MCA, which so far has not been documented in the circum-Arctic nor the Northern Hemisphere reconstructions^1^,^4^,^5^.

Multi-decadal climatic variations during the last millennium may arise either from internal climate variability, linked to ocean-atmosphere-sea ice interactions, or from climatic response to volcanic and/or solar forcing. Because MCA temperature variations at the hemispheric scale do not coincide with regional climate variations (Fig. 2), these regional changes may be due to internal variability^34^. A long-lasting, indirect impact of volcanic eruptions on regional climate variability has been suggested through increased bidecadal variations in the North Atlantic gyre, with Labrador/Baffin Bay sea ice feedbacks^35^. Such oceanic circulation variations can drive reverse sea ice patterns in the Disko region compared to south-eastern Greenland/Iceland. In Disko^30^, a gradual decline in sea ice concentration is documented during the last millennium, acting as a positive feedback on atmospheric temperature. Conversely, in south-eastern Greenland and Iceland a maximum sea ice concentration is recorded during the LIA^30^,^33^,^36^. Several recent studies have identified solar and volcanic forcing as important triggers for sea-ice variability over the last millennium^36^,^37^.

**Figure 3:**
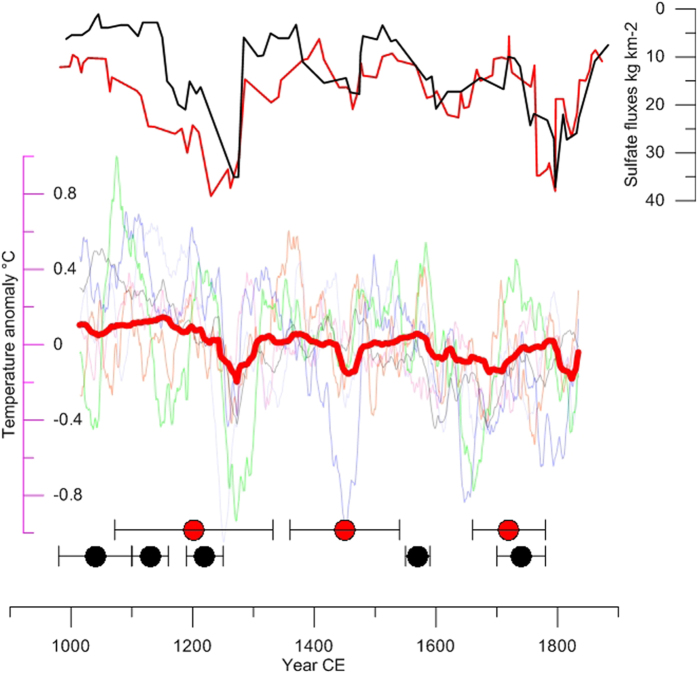
Volcanic forcing, simulated Greenland annual temperature anomaly, and moraine records. Upper panel: Northern Hemisphere volcanic sulfate fluxes 5 year running mean; red after ref. 22; black after *sup* ref 51 (extended data Table S5). Middle panel: Red, PMIP-3 ensemble mean temperature over Greenland (65–15° W, 60–83° N) (extended Table S5); CCSM4 = blue; Fgoals = pink; IPSL = green; GISS = black: MPI = grey BCC = orange. Anomalies are with respect to the 1000–1850 CE period. Lower panel: Red circles are the moraine record at Disko; Black circles are the moraine record in Baffin Bay^19^. Error bars are moraine age uncertainties related to standard deviation, analytical and production rate uncertainties. The figure was generated with Grapher 11, Golden Software. www.goldensoftxware.com.

However, exploring the synchronicity of external forcings and the Disko moraine record, we show a good correspondence between both records, suggesting that internal variability is combined with other factors. Indeed, we show that M2 (~1200 CE) is contemporaneous with a 50-year period of high volcanic activity with repeated large eruptions, including the 1257 Samalas event (Fig. 2). M3 (~1450 CE) is concomitant with both a major nameless volcanic eruption (1458 CE) and the Spörer Minimum. By contrast, M4 formed towards the end of the LIA (~1720 CE), is associated with low volcanic activity (Fig. 2), but may correspond to the Maunder Minimum (1645–1715 CE). MCA moraines from Baffin Bay^19^ are also associated with the Oort Minimum and with a cluster of several successive volcanic eruptions in the first two centuries of the last millennium (Figs 2 and 3). LIA moraines also coincide with volcanic eruptions or solar minima, except for a moraine deposited in 1570 ± 20 yr CE.

We now investigate simulated temperature changes from the PMIP-3 ensemble (Extended data Table 5). In response to solar and volcanic forcing, the ensemble results display mild conditions in the region during the MCA until 1250 CE and cold multi-decadal periods during the LIA (Fig. 3). The coherence between external forcing, climate simulations and the formation of M3 and M4 moraines suggests that LIA glacier advances are likely a response to externally-forced temperature minima at 1450 CE and 1700 CE, respectively (Fig. 3). However, the PMIP-3 simulations are not consistent with the moraine record during the MCA, and the compelling evidence for cool conditions during the MCA. While PMIP-3 ensemble simulations depict 0.5 °C post-Samalas regional cooling after 1250 CE with quite large differences between models, (Fig. 3), the mechanisms of glacier advance following a positive mass balance requires an earlier occurrence of colder conditions to explain the formation of M2 at Disko and MCA moraines in Baffin Bay.

We finally explore the reasons for such model-data discrepancy. A recent study^38^ revealed that microphysical processes are crucial for correctly simulating the relationship between volcanic aerosols and their radiative forcing, and their subsequent climate impact. PMIP-3 models likely overestimate the radiative forcing and climatic responses of large eruptions, due to simplistic parameterization of aerosol optical depth. As a result, several successive mid-intensity volcanic eruptions may have stronger climatic impacts than a single very large eruption. New ice core data show more occurrences of volcanic eruptions between 1000 and 1200 CE^24^ than previously considered in PMIP-3 simulations^21^ (Fig. 3). The model-data mismatch may therefore result from inadequate representation of volcanic forcing, or inadequate excitation of regional climate responses affecting specifically the west Arctic in coupled climate models (including ocean and sea ice processes), or from a dominant role of (unpredictable) internal variability. Assimilating information from Western Arctic glacier fluctuation in climate simulations with more realistic volcanic forcing would help to understand the moraine record. Altogether, our moraine data suggests that West “Green land” evolved towards an “ice land” at the end of the MCA, with similar extents to that of the ensuing LIA.

## Methods

### The chronology of Lyngmarksbræen glacier fluctuations

The moraines from a small (1.75 km^2^) outlet of Lyngmarksbræen ice cap, Disko Island, West Greenland were used to estimate past glacial extents. Preserved moraines represent the minimum number of fluctuations of the glacier in the past. The behaviour of the glacier between two successive moraine ridges is unknown. The glacier may have strongly retreated between two successive moraines. We assume that moraine ridges were formed at the time when the glacier is in, or close to, equilibrium with the prevailing climate. The response time of the glacier is assumed to be less than the uncertainty of the dates provided in this paper due to the small size and the steep slope. The ages provided by ^36^Cl of boulders on moraine ridges represent the onset of the retreat.

Samples (n = 14) were taken from boulders on the top of moraine and moraine remnants. All samples were collected with a manual jackhammer from broadly horizontal or sub-horizontal surfaces. Sample locations and elevations were recorded using a handheld GPS, sample thickness and topographic shielding (horizon line) were measured using a clinometer (Table S1).

The physical and chemical sample preparation for ^36^Cl extraction was performed at CEREGE (Aix-en-Provence, France). Whole rock samples were crushed and sieved. A split of bulk rock was kept aside for major and trace element analysis performed at the Service d’Analyse des Roches et des Minéraux (SARM, Nancy, France) (Table S2). The 0.25–0.50 mm grain size fraction was leached in a dilute HF/HNO3 acid mixture to remove about 20% of the initial sample mass in order to decontaminate from atmospheric ^36^Cl and from potentially Cl-rich groundmass^23^. After this pretreatment, 2 g aliquots of the remaining solid sample grains were taken to quantify the concentrations of the target elements for spallogenic/muogenic production of ^36^Cl (Ca, K, Ti, Fe) by ICP-OES at SARM (Table S3). The ^36^Cl extraction then follows the protocol outlined in ref. 39. Before total dissolution, a ^35^Cl-enriched spike (~99%) was added to each sample for isotope dilution^39^. Two procedural blanks were performed. Concentrations of ^36^Cl and Cl were determined by isotope dilution AMS measurements at ASTER-CEREGE (Table S4). ^36^Cl/^35^Cl ratios were determined by normalizing to a ^36^Cl standard prepared by K. Nishiizumi^41^. The stable ratio ^35^Cl/^37^Cl was also normalized to this standard, assuming a natural ratio of 3.127.

Final exposure ages were calculated using the Excel ^36^Cl age calculation spreadsheet by ref. 23 based on sample compositions, corrections for sample thickness, and topographic shielding. Calculations were done with the time-invariant “St” scaling^24^ and two alternative production rate sets from refs 40 and 42 (hereafter “Schimmelpfennig” production rate set) and from ref. 43 (“Marrero” production rate set), respectively, with the results from both being presented in the Extended data Table S4.

In case of the “Schimmelpfennig” production rate set, the parameters, referenced to sea level and high latitude (SLHL), are: 42.2 ± 4.8 atoms ^36^Cl (g Ca)^−1^ yr^−1^ for spallation of Ca^40^, 148.1 ± 7.8 atoms ^36^Cl (g K)^−1^ yr^−1^ for spallation of K^42^, 13 ± 3 atoms ^36^Cl (g Ti)^−1^ yr^−1^ for spallation of Ti^44^, 1.9 ± 0.2 atoms ^36^Cl (g Fe)^−1^ yr^−1^ for spallation of Fe^45^, and 626 neutrons (g air)^−1^ yr^−1^ for the production rate of epithermal neutrons from fast neutrons in the atmosphere at the land/atmosphere interface^46^.

In case of the “Marrero” production rate set, the SLHL parameters are 52.2 ± 5.2 atoms ^36^Cl (g Ca)^−1^ yr^−1^ for spallation of Ca^43^, 150 ± 15 atoms ^36^Cl (g K)^−1^ yr^−1^ for spallation of K^43^, 3.8 atoms ^36^Cl (g Ti)^−1^ yr^−1^ for spallation of Ti^43^,^47^ 1.9 ± 0.2 atoms ^36^Cl (g Fe)^−1^ yr^−1^ for spallation of Fe^45^, and 696 ± 185 neutrons (g air)^−1^ yr^−1^ for the production rate of epithermal neutrons from fast neutrons in the atmosphere at the land/atmosphere interface^43^.

In both cases, the high-energy neutron attenuation length 160 g cm^−2^ was applied. Parameters for ^36^Cl production from slow muon capture by Ca and K were not changed in the originally published Excel spreadsheet^23^.

The ages presented in the main text are based on the “Schimmelpfennig” production rate set. Our conclusions are robust with respect to discrepancies in production rates causing ages younger by ~12% or less, when using the “Marrero” production rate set. These differences are within the global errors of the moraine ages. Using the recently published CRONUScalc program^48^ for the ^36^Cl age calculations, yields results that are insignificantly different from the ages presented here (~2%).

No corrections for erosion were applied to the ages, because little field evidence was found for weathering of the sampled boulder surfaces. In addition, due to the short exposure duration of the boulders that were deposited during the last millennium, the correction for a hypothetical erosion rate of 1 mm yr^−1^ would have an insignificant impact on those moraine ages (ages would be older by <1%). A potential effect from snow cover on the presented ages was not accounted for in concert with the ^10^Be ages from Baffin Bay^19^, which we compare our ^36^Cl ages to.

## Additional Information

**How to cite this article**: Jomelli, V. *et al.* Paradoxical cold conditions during the medieval climate anomaly in the Western Arctic. *Sci. Rep.* **6**, 32984; doi: 10.1038/srep32984 (2016).

## Supplementary Material

Supplementary Information

Supplementary Tables
