# Supplementary material for: Paradoxical cold conditions during the medieval climate anomaly in the Western Arctic
Source: Sci Rep. 2016 Sep 9;6:32984. doi: 10.1038/srep32984 (PMC5016737; doi:10.1038/srep32984)
Supplement: Supplementary Information [file srep32984-s1.doc]

**Supplementary Online Material**

**Paradoxical cold conditions during the medieval climate anomaly in the Western Arctic**

**Vincent Jomelli^*^, Timothy Lane, Vincent Favier, Valerie Masson-Delmotte, Didier Swingedouw, Vincent Rinterknecht, Irene Schimmelpfennig, Daniel Brunstein, Deborah Verfaillie, Kathryn Adamson, Laëtitia Leanni, Fatima Mokadem, ASTER Team**

The following pages include:

Figure S1

The list of extended tables 1-5

References


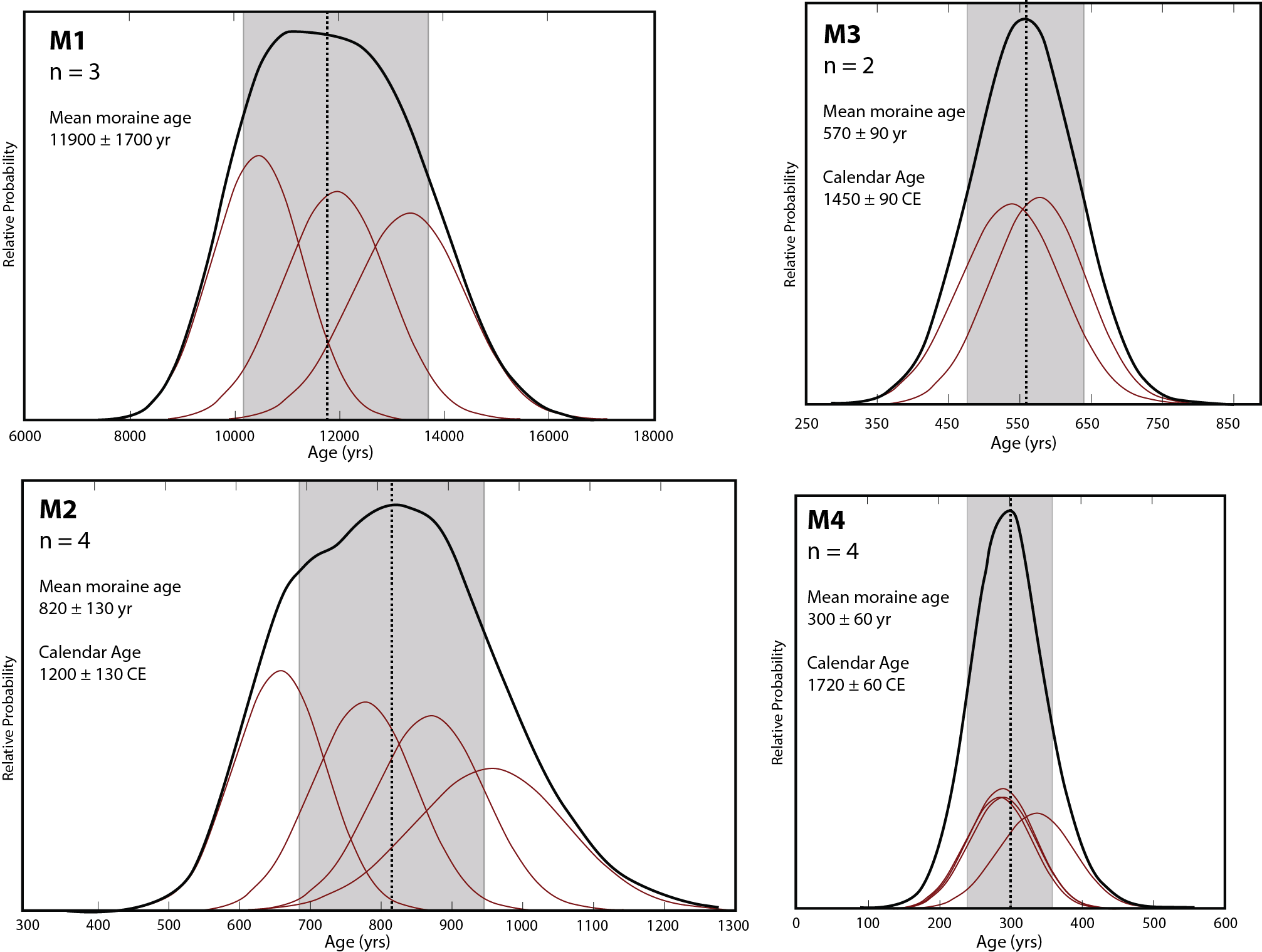


**Extended Data Figure 1.** Summed probability plots the ^36^Cl surface exposure ages for each moraine based on the “Schimmelpfennig” production rate set (Methods). Red gaussians are the individual boulder ages with 1σ analytical uncertainty only. Dashed vertical lines are the arithmetic means. Grey bands represent the 1σ errors including standard deviation, analytical and production rate errors.

**Table S1:** Sample locations and sample-specific information.

**Table S2:** Chemical compositions of two bulk rock samples before acid etching. Analysis performed at the SARM-CRPG (Nancy, France) by ICP-OES (major elements), ICP-MS (trace element), atomic absorption (Li), colorimetry (B) and spectrophotometry (Cl). The

**Table S3:** Concentrations of the ^36^Cl target elements, determined in splits taken from the samples after the acid etching to remove ~20% of the samples. Analysis performed at the SARM-CRPG (Nancy, France) by ICP-OES. Concentrations of the target element Cl

**Table S4:** ^36^Cl dating results, including measured ^35^Cl/^37^Cl and ^36^Cl/^35^Cl ratios, inferred ^36^Cl and Cl concentrations, individual sample ages, and landform mean ages. Data for the two procedural blanks (DIS-Bk_01 and DIS-Bk_02) are also included. DIS-Bk_

**Table S5:** Climate models decription

**Supplementary References**

1. Schimmelpfennig, I., *et al.* Calibration of cosmogenic ^36^Cl production rates from Ca and K spallation in lava flows from Mt. Etna (38°N, Italy) and Payun Matru (36°S, Argentina). *Geochimica et Cosmochimica Acta* **75**, 2611-2632 (2011).
2. Ivy-Ochs, S., *et al*. Initial results from isotope dilution for Cl and 36Cl measurements at the PSI/ETH Zurich AMS facility. *Nuclear Instruments and Methods in Physics* *Research Section B* **223** 623- 627, (2004).
3. Sharma, P., *et al.* Development of ^36^Cl standards for AMS. *Nuclear Instruments and Methods in Physics Research Section B* **52**, 410-415 (1990).
4. Schimmelpfennig, I., *et al.* 36Cl production rate from K‐spallation in the European Alps (Chironico landslide, Switzerland). *Journal of Quaternary Science* **29**, 407-413 (2014).
5. Marrero, S.M., Phillips , F.M., Caffee, M.W., & Gosse, J.C. CRONUS-Earth cosmogenic ^36^Cl calibration. *Quaternary Geochronology* **31**, 199-219 (2016).
6. Fink D., Vogt S. & Hotchkis M. Cross-sections for ^36^Cl from Ti at E_p_ = 35–150 MeV: applications to in-situ exposure dating. *Nucl. Instrum. Meth. Phys. Res.* *Sect. B* **172**, 861–866 (2000).
7. Stone J.O., Fifield, K., & Vasconcelos, P. Terrestrial chlorine-36 production from spallation of iron. Abstract of 10th International Conference on Accelerator Mass Spectrometry. September 5-10, 2005, Berkeley, USA. <http://llnl.confex.com/llnl/ams10/techprogram/P1397.HTM> (2005).
8. Phillips, F.M., Stone, W.D., & Fabryka-Martin, J.T. An improved approach to calculating low-energy cosmic-ray neutron fluxes near the land/atmosphere interface. *Chem. Geol*. **175**, 689–701 (2001).
9. Masarik, J. Numerical simulation of in-situ produced cosmogenic nuclides. *Geochimica Cosmochimica Acta* **66**, A491. (2002).
10. Marrero, S.M. *et al*. Cosmogenic nuclide systematics and the CRONUScalc program. *Quaternary Geochronology* **31**, 160-187 (2016).
11. Muscheler, R., *et al.* Solar activity during the last 1000 yr inferred from radionuclide records. *Quat. Sci.Rev*. **26**, 82-97 (2007).
12. Gao, C., Robock, A., & Ammann, C. Volcanic forcing of climate over the past 1500 years. An improved ice core‐based index for climate models. *Journal of Geophysical Research: Atmospheres* **113**, 1984–2012 (2008).
13. Crowley, T.J.& Unterman, M.B. Technical details concerning development of a 1200-yr proxy index for global volcanism. *Earth System Science Data*, **5**, 187-197 (2013).
14. Landrum, L. *et al.* Last Millennium Climate and Its Variability in CCSM4. *Journal of Climate* **26**, 1085–1111 [doi/abs/10.1175/JCLI-D-11-00326.1](http://journals.ametsoc.org/doi/abs/10.1175/JCLI-D-11-00326.1) (2013).
15. Jungclaus, J.H., Lohmann, K. & Zanchettin, D., Enhanced 20th century heat transfer to the Arctic simulated in the context of climate variations over the last millennium. *Climate of the Past Discussions* **10,** 2895–2924. <http://www.clim-past-discuss.net/10/2895/2014/> (2014).
16. Schmidt, G. a. *et al*. Climate forcing reconstructions for use in PMIP simulations of the Last Millennium (v1.1). *Geoscientific Model Development* **5**, 185–191. (2012).
17. Zhang, J., & Wu, T. The impact of external forcings on climate during the past millennium: Results from transient simulation with BCC_CSM1. 1, *in* *Proceedings EGU General Assembly Conference Abstract* **14**, 448. (2012).
18. Zhou, T. et al. A comparison of the Medieval Warm Period, Little Ice Age and 20th century warming simulated by the FGOALS climate system model. *Chinese Science Bulletin,* **56**, 3028–3041 (2011).
